# Ethyl 6-chloro-2-oxo-4-phenyl-1,2-dihydro­quinoline-3-carboxyl­ate

**DOI:** 10.1107/S1600536809045425

**Published:** 2009-11-04

**Authors:** F. Nawaz Khan, Suganya Mittal, Soheil Anjum, Venkatesha R. Hathwar, Seik Weng Ng

**Affiliations:** a Chemistry Division, School of Science and Humanities, VIT University, Vellore 632 014, Tamil Nadu, India; b Solid State and Structural Chemistry Unit, Indian Institute of Science, Bangalore 560 012, Karnataka, India; c Department of Chemistry, University of Malaya, 50603 Kuala Lumpur, Malaysia

## Abstract

In the title compound, C_18_H_14_ClNO_3_, the dihydro­quinolin-2-one ring system is almost planar (r.m.s. deviation = 0.033 Å). The carboxyl­ate plane and the phenyl group are twisted away from the dihydro­quinolin-2-one ring system by 50.3 (1) and 64.9 (1)°, respectively. In the crystal structure, inversion-related mol­ecules form *R*_2_^2^(8) dimers *via* pairs of N—H⋯O hydrogen bonds.

## Related literature

For crystal structures of related compounds, see: Baumer *et al.* (2001 ▶); Subashini *et al.* (2009 ▶).
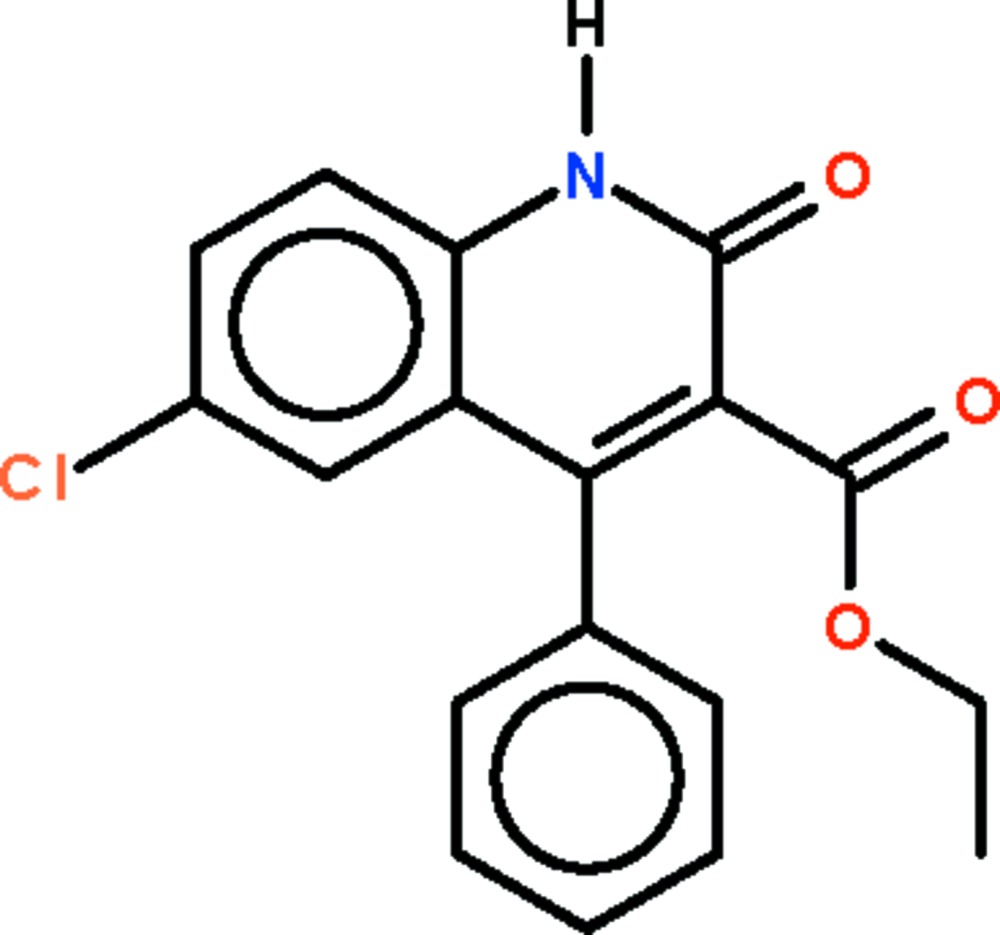


## Experimental

### 

#### Crystal data

C_18_H_14_ClNO_3_
*M* *_r_* = 327.75
Monoclinic, 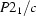

*a* = 10.176 (1) Å
*b* = 15.629 (2) Å
*c* = 11.282 (1) Å
β = 115.463 (1)°
*V* = 1619.9 (3) Å^3^
*Z* = 4
Mo *K*α radiation
μ = 0.25 mm^−1^
*T* = 290 K
0.35 × 0.31 × 0.23 mm

#### Data collection

Bruker SMART CCD area-detector diffractometer
Absorption correction: multi-scan (*SADABS*; Sheldrick, 1996 ▶) *T*_min_ = 0.918, *T*_max_ = 0.945
13600 measured reflections
3699 independent reflections
2906 reflections with *I* > 2σ(*I*)
*R*_int_ = 0.022

#### Refinement

*R*[*F* ^2^ > 2σ(*F* ^2^)] = 0.046
*wR*(*F* ^2^) = 0.129
*S* = 1.00
3699 reflections
213 parameters
H atoms treated by a mixture of independent and constrained refinement
Δρ_max_ = 0.23 e Å^−3^
Δρ_min_ = −0.20 e Å^−3^

### 

Data collection: *SMART* (Bruker, 2004 ▶); cell refinement: *SAINT* (Bruker, 2004 ▶); data reduction: *SAINT*; program(s) used to solve structure: *SHELXS97* (Sheldrick, 2008 ▶); program(s) used to refine structure: *SHELXL97* (Sheldrick, 2008 ▶); molecular graphics: *X-SEED* (Barbour, 2001 ▶); software used to prepare material for publication: *publCIF* (Westrip, 2009 ▶).

## Supplementary Material

Crystal structure: contains datablocks global, I. DOI: 10.1107/S1600536809045425/ci2958sup1.cif

Structure factors: contains datablocks I. DOI: 10.1107/S1600536809045425/ci2958Isup2.hkl

Additional supplementary materials:  crystallographic information; 3D view; checkCIF report

## Figures and Tables

**Table 1:** Hydrogen-bond geometry (Å, °)

*D*—H⋯*A*	*D*—H	H⋯*A*	*D*⋯*A*	*D*—H⋯*A*
N1—H1⋯O1^i^	0.88 (2)	1.89 (2)	2.763 (2)	178 (2)

Symmetry code: (i) 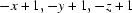.

**Fig. 1.**
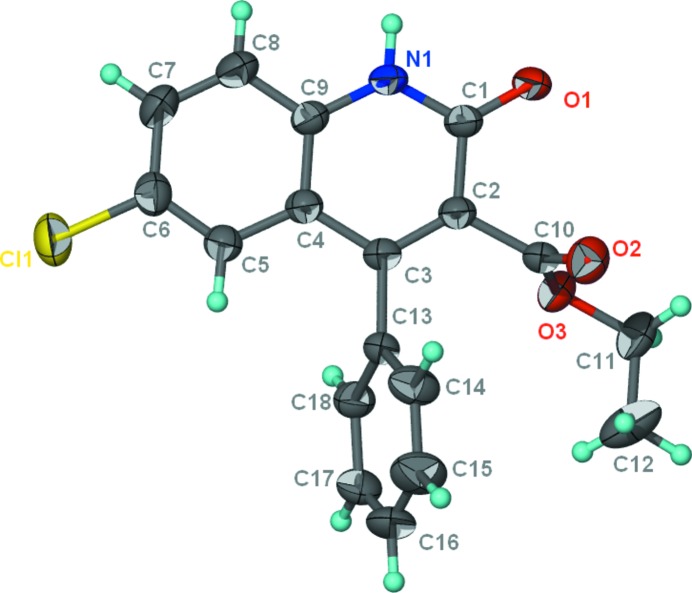
Displacement ellipsoid plot (Barbour, 2001) of C18H14ClNO3 at the 50% probability level. H atoms are drawn as spheres of arbitrary radius.

## supplementary crystallographic information

### Experimental

(2-Amino-5-chlorophenyl)(phenyl)methanone (1 mmol) and diethyl malonate (1.2 mmol) along with a catalytic amount of piperidine were heated at 453 K; the reaction was monitored by TLC. After completion, the reaction mixture was poured into the water. The organic product was extracted with ethyl acetate. The crude product was then purified by silica-gel column chromatography, with petroleum ether and ethyl acetate as eluant. Single crystals were obtained by recrystallization from ethyl acetate.

### Refinement

C-bound H-atoms were placed in calculated positions (C-H = 0.93–0.97 Å) and were included in the refinement in the riding model approximation, with *U_iso_*(H) set to 1.2*U_eq_*(C). The amino H-atom was located in a difference Fourier map, and was freely refined without any restraint.

### Crystal data

**Table:**

C_18_H_14_ClNO_3_	*F*(000) = 680
*M**_r_* = 327.75	*D*_x_ = 1.344 Mg m^−^^3^
Monoclinic, *P*2_1_/*c*	Mo *K*α radiation, λ = 0.71073 Å
Hall symbol: -P 2ybc	Cell parameters from 1123 reflections
*a* = 10.176 (1) Å	θ = 2.9–20.7°
*b* = 15.629 (2) Å	µ = 0.25 mm^−^^1^
*c* = 11.282 (1) Å	*T* = 290 K
β = 115.463 (1)°	Block, colourless
*V* = 1619.9 (3) Å^3^	0.35 × 0.31 × 0.23 mm
*Z* = 4	

### Data collection

**Table:**

Bruker SMART CCD area-detector diffractometer	3699 independent reflections
Radiation source: fine-focus sealed tube	2906 reflections with *I* > 2σ(*I*)
graphite	*R*_int_ = 0.022
φ and ω scans	θ_max_ = 27.5°, θ_min_ = 2.2°
Absorption correction: multi-scan (*SADABS*; Sheldrick, 1996)	*h* = −13→13
*T*_min_ = 0.918, *T*_max_ = 0.945	*k* = −20→19
13600 measured reflections	*l* = −14→14

### Refinement

**Table:**

Refinement on *F*^2^	Primary atom site location: structure-invariant direct methods
Least-squares matrix: full	Secondary atom site location: difference Fourier map
*R*[*F*^2^ > 2σ(*F*^2^)] = 0.046	Hydrogen site location: inferred from neighbouring sites
*wR*(*F*^2^) = 0.129	H atoms treated by a mixture of independent and constrained refinement
*S* = 1.00	*w* = 1/[σ^2^(*F*_o_^2^) + (0.0664*P*)^2^ + 0.4015*P*] where *P* = (*F*_o_^2^ + 2*F*_c_^2^)/3
3699 reflections	(Δ/σ)_max_ = 0.001
213 parameters	Δρ_max_ = 0.23 e Å^−^^3^
0 restraints	Δρ_min_ = −0.20 e Å^−^^3^

### Special details

**Table:**

Geometry. All e.s.d.'s (except the e.s.d. in the dihedral angle between two l.s. planes) are estimated using the full covariance matrix. The cell e.s.d.'s are taken into account individually in the estimation of e.s.d.'s in distances, angles and torsion angles; correlations between e.s.d.'s in cell parameters are only used when they are defined by crystal symmetry. An approximate (isotropic) treatment of cell e.s.d.'s is used for estimating e.s.d.'s involving l.s. planes.

### Fractional atomic coordinates and isotropic or equivalent isotropic displacement parameters (Å^2^)

**Table:**

	*x*	*y*	*z*	*U*_iso_*/*U*_eq_	
Cl1	0.54343 (6)	0.02895 (3)	0.68514 (6)	0.0743 (2)	
N1	0.46268 (16)	0.38491 (9)	0.50054 (15)	0.0526 (4)	
H1	0.527 (2)	0.4235 (14)	0.546 (2)	0.066 (6)*	
O1	0.33674 (13)	0.49099 (7)	0.36051 (13)	0.0569 (3)	
O2	0.13633 (14)	0.40486 (9)	0.08985 (13)	0.0605 (4)	
O3	0.00732 (12)	0.38779 (8)	0.20662 (11)	0.0487 (3)	
C1	0.35167 (17)	0.41406 (10)	0.38896 (17)	0.0454 (4)	
C2	0.25139 (17)	0.34892 (10)	0.30798 (15)	0.0404 (3)	
C3	0.26581 (16)	0.26477 (10)	0.34062 (15)	0.0384 (3)	
C4	0.38454 (16)	0.23795 (10)	0.46323 (16)	0.0405 (4)	
C5	0.40613 (18)	0.15305 (11)	0.50898 (16)	0.0450 (4)	
H5	0.3452	0.1098	0.4580	0.054*	
C6	0.5166 (2)	0.13411 (12)	0.62836 (19)	0.0530 (4)	
C7	0.6110 (2)	0.19671 (14)	0.7067 (2)	0.0648 (5)	
H7	0.6853	0.1826	0.7880	0.078*	
C8	0.5932 (2)	0.27920 (13)	0.6629 (2)	0.0634 (5)	
H8	0.6566	0.3213	0.7144	0.076*	
C9	0.48116 (18)	0.30096 (11)	0.54190 (17)	0.0465 (4)	
C10	0.12728 (17)	0.38317 (10)	0.18746 (15)	0.0411 (4)	
C11	−0.1227 (2)	0.42135 (14)	0.09932 (19)	0.0620 (5)	
H11A	−0.0961	0.4681	0.0573	0.074*	
H11B	−0.1882	0.4437	0.1338	0.074*	
C12	−0.1985 (2)	0.3536 (2)	0.0004 (2)	0.0894 (8)	
H12A	−0.2907	0.3749	−0.0625	0.134*	
H12B	−0.2140	0.3044	0.0438	0.134*	
H12C	−0.1398	0.3378	−0.0437	0.134*	
C13	0.15864 (17)	0.20196 (10)	0.25160 (15)	0.0400 (3)	
C14	0.1482 (2)	0.18833 (13)	0.12633 (19)	0.0605 (5)	
H14	0.2126	0.2156	0.1001	0.073*	
C15	0.0423 (3)	0.13422 (15)	0.0401 (2)	0.0782 (7)	
H15	0.0358	0.1254	−0.0438	0.094*	
C16	−0.0525 (3)	0.09389 (13)	0.0781 (2)	0.0717 (6)	
H16	−0.1231	0.0574	0.0202	0.086*	
C17	−0.0437 (2)	0.10699 (12)	0.2011 (2)	0.0619 (5)	
H17	−0.1087	0.0796	0.2264	0.074*	
C18	0.06135 (19)	0.16075 (11)	0.28790 (17)	0.0499 (4)	
H18	0.0667	0.1693	0.3714	0.060*	

### Atomic displacement parameters (Å^2^)

**Table:**

	*U*^11^	*U*^22^	*U*^33^	*U*^12^	*U*^13^	*U*^23^
Cl1	0.0758 (4)	0.0599 (3)	0.0810 (4)	0.0093 (2)	0.0277 (3)	0.0260 (3)
N1	0.0408 (8)	0.0370 (8)	0.0583 (9)	−0.0040 (6)	0.0007 (7)	−0.0097 (7)
O1	0.0437 (7)	0.0348 (6)	0.0701 (8)	−0.0018 (5)	0.0034 (6)	−0.0062 (6)
O2	0.0600 (8)	0.0724 (9)	0.0500 (7)	0.0001 (7)	0.0246 (6)	0.0108 (6)
O3	0.0396 (6)	0.0591 (7)	0.0424 (6)	0.0039 (5)	0.0127 (5)	0.0059 (5)
C1	0.0350 (8)	0.0384 (9)	0.0528 (9)	−0.0012 (6)	0.0095 (7)	−0.0072 (7)
C2	0.0370 (8)	0.0392 (8)	0.0407 (8)	−0.0041 (6)	0.0126 (7)	−0.0051 (6)
C3	0.0355 (8)	0.0392 (8)	0.0395 (8)	−0.0040 (6)	0.0152 (7)	−0.0057 (6)
C4	0.0362 (8)	0.0400 (8)	0.0427 (8)	−0.0010 (6)	0.0145 (7)	−0.0050 (7)
C5	0.0426 (9)	0.0419 (9)	0.0486 (9)	−0.0011 (7)	0.0178 (7)	−0.0018 (7)
C6	0.0515 (10)	0.0491 (10)	0.0568 (10)	0.0070 (8)	0.0218 (9)	0.0085 (8)
C7	0.0559 (11)	0.0654 (13)	0.0508 (10)	0.0066 (9)	0.0018 (9)	0.0056 (9)
C8	0.0521 (11)	0.0556 (11)	0.0571 (11)	−0.0020 (9)	−0.0007 (9)	−0.0076 (9)
C9	0.0382 (8)	0.0423 (9)	0.0477 (9)	0.0008 (7)	0.0079 (7)	−0.0049 (7)
C10	0.0413 (8)	0.0349 (8)	0.0405 (8)	−0.0062 (6)	0.0115 (7)	−0.0048 (6)
C11	0.0442 (10)	0.0760 (14)	0.0559 (11)	0.0132 (9)	0.0121 (9)	0.0111 (10)
C12	0.0518 (13)	0.133 (2)	0.0645 (14)	−0.0043 (14)	0.0072 (11)	−0.0217 (15)
C13	0.0406 (8)	0.0347 (8)	0.0398 (8)	−0.0046 (6)	0.0127 (7)	−0.0029 (6)
C14	0.0770 (13)	0.0589 (12)	0.0531 (10)	−0.0188 (10)	0.0352 (10)	−0.0146 (9)
C15	0.1034 (18)	0.0738 (15)	0.0521 (12)	−0.0204 (13)	0.0283 (12)	−0.0255 (10)
C16	0.0731 (14)	0.0518 (12)	0.0663 (13)	−0.0210 (10)	0.0073 (11)	−0.0175 (10)
C17	0.0506 (11)	0.0514 (11)	0.0736 (13)	−0.0162 (8)	0.0171 (10)	0.0005 (9)
C18	0.0511 (10)	0.0508 (10)	0.0450 (9)	−0.0105 (8)	0.0181 (8)	−0.0008 (7)

### Geometric parameters (Å, °)

**Table:**

Cl1—C6	1.7424 (19)	C8—C9	1.393 (2)
N1—C1	1.359 (2)	C8—H8	0.93
N1—C9	1.378 (2)	C11—C12	1.490 (3)
N1—H1	0.88 (2)	C11—H11A	0.97
O1—C1	1.237 (2)	C11—H11B	0.97
O2—C10	1.193 (2)	C12—H12A	0.96
O3—C10	1.330 (2)	C12—H12B	0.96
O3—C11	1.454 (2)	C12—H12C	0.96
C1—C2	1.453 (2)	C13—C18	1.382 (2)
C2—C3	1.357 (2)	C13—C14	1.388 (2)
C2—C10	1.501 (2)	C14—C15	1.386 (3)
C3—C4	1.453 (2)	C14—H14	0.93
C3—C13	1.490 (2)	C15—C16	1.367 (3)
C4—C9	1.405 (2)	C15—H15	0.93
C4—C5	1.406 (2)	C16—C17	1.367 (3)
C5—C6	1.365 (2)	C16—H16	0.93
C5—H5	0.93	C17—C18	1.381 (3)
C6—C7	1.389 (3)	C17—H17	0.93
C7—C8	1.365 (3)	C18—H18	0.93
C7—H7	0.93		
			
C1—N1—C9	124.83 (14)	O2—C10—C2	124.65 (15)
C1—N1—H1	115.4 (14)	O3—C10—C2	110.23 (13)
C9—N1—H1	119.8 (14)	O3—C11—C12	111.10 (18)
C10—O3—C11	117.05 (13)	O3—C11—H11A	109.4
O1—C1—N1	121.80 (15)	C12—C11—H11A	109.4
O1—C1—C2	122.91 (15)	O3—C11—H11B	109.4
N1—C1—C2	115.28 (15)	C12—C11—H11B	109.4
C3—C2—C1	122.95 (15)	H11A—C11—H11B	108.0
C3—C2—C10	122.83 (14)	C11—C12—H12A	109.5
C1—C2—C10	114.18 (14)	C11—C12—H12B	109.5
C2—C3—C4	119.12 (14)	H12A—C12—H12B	109.5
C2—C3—C13	119.56 (14)	C11—C12—H12C	109.5
C4—C3—C13	121.31 (14)	H12A—C12—H12C	109.5
C9—C4—C5	118.23 (15)	H12B—C12—H12C	109.5
C9—C4—C3	117.95 (14)	C18—C13—C14	118.65 (15)
C5—C4—C3	123.80 (14)	C18—C13—C3	121.14 (14)
C6—C5—C4	119.94 (16)	C14—C13—C3	120.06 (14)
C6—C5—H5	120.0	C15—C14—C13	120.29 (18)
C4—C5—H5	120.0	C15—C14—H14	119.9
C5—C6—C7	121.72 (17)	C13—C14—H14	119.9
C5—C6—Cl1	119.97 (15)	C16—C15—C14	120.11 (19)
C7—C6—Cl1	118.31 (15)	C16—C15—H15	119.9
C8—C7—C6	119.16 (18)	C14—C15—H15	119.9
C8—C7—H7	120.4	C15—C16—C17	120.18 (18)
C6—C7—H7	120.4	C15—C16—H16	119.9
C7—C8—C9	120.70 (18)	C17—C16—H16	119.9
C7—C8—H8	119.6	C16—C17—C18	120.24 (19)
C9—C8—H8	119.6	C16—C17—H17	119.9
N1—C9—C8	119.95 (16)	C18—C17—H17	119.9
N1—C9—C4	119.80 (15)	C17—C18—C13	120.53 (17)
C8—C9—C4	120.24 (16)	C17—C18—H18	119.7
O2—C10—O3	125.10 (15)	C13—C18—H18	119.7
			
C9—N1—C1—O1	176.16 (17)	C7—C8—C9—C4	0.1 (3)
C9—N1—C1—C2	−2.6 (3)	C5—C4—C9—N1	−179.57 (16)
O1—C1—C2—C3	−178.45 (16)	C3—C4—C9—N1	−1.1 (2)
N1—C1—C2—C3	0.3 (2)	C5—C4—C9—C8	−1.2 (3)
O1—C1—C2—C10	−0.6 (2)	C3—C4—C9—C8	177.35 (16)
N1—C1—C2—C10	178.23 (15)	C11—O3—C10—O2	0.5 (2)
C1—C2—C3—C4	1.4 (2)	C11—O3—C10—C2	178.93 (14)
C10—C2—C3—C4	−176.30 (14)	C3—C2—C10—O2	−104.7 (2)
C1—C2—C3—C13	−179.62 (14)	C1—C2—C10—O2	77.4 (2)
C10—C2—C3—C13	2.7 (2)	C3—C2—C10—O3	76.87 (19)
C2—C3—C4—C9	−1.0 (2)	C1—C2—C10—O3	−101.02 (16)
C13—C3—C4—C9	−179.99 (14)	C10—O3—C11—C12	82.5 (2)
C2—C3—C4—C5	177.38 (15)	C2—C3—C13—C18	−111.20 (19)
C13—C3—C4—C5	−1.6 (2)	C4—C3—C13—C18	67.7 (2)
C9—C4—C5—C6	1.5 (2)	C2—C3—C13—C14	64.3 (2)
C3—C4—C5—C6	−176.91 (15)	C4—C3—C13—C14	−116.78 (19)
C4—C5—C6—C7	−0.8 (3)	C18—C13—C14—C15	−0.2 (3)
C4—C5—C6—Cl1	179.98 (13)	C3—C13—C14—C15	−175.75 (19)
C5—C6—C7—C8	−0.4 (3)	C13—C14—C15—C16	−0.1 (4)
Cl1—C6—C7—C8	178.89 (17)	C14—C15—C16—C17	0.3 (4)
C6—C7—C8—C9	0.7 (3)	C15—C16—C17—C18	−0.3 (4)
C1—N1—C9—C8	−175.35 (18)	C16—C17—C18—C13	0.1 (3)
C1—N1—C9—C4	3.1 (3)	C14—C13—C18—C17	0.2 (3)
C7—C8—C9—N1	178.5 (2)	C3—C13—C18—C17	175.69 (17)

### Hydrogen-bond geometry (Å, °)

**Table:**

*D*—H···*A*	*D*—H	H···*A*	*D*···*A*	*D*—H···*A*
N1—H1···O1^i^	0.88 (2)	1.89 (2)	2.763 (2)	178 (2)
C11—H11A···O2^ii^	0.97	2.51	3.420 (3)	157
C17—H17···O1^iii^	0.93	2.51	3.299 (3)	143
C18—H18···O2^iv^	0.93	2.53	3.317 (2)	142

Symmetry codes: (i) −*x*+1, −*y*+1, −*z*+1; (ii) −*x*, −*y*+1, −*z*; (iii) −*x*, *y*−1/2, −*z*+1/2; (iv) *x*, −*y*+1/2, *z*+1/2.
